# Mobile Apps for People With Rare Diseases: Review and Quality Assessment Using Mobile App Rating Scale

**DOI:** 10.2196/36691

**Published:** 2022-07-26

**Authors:** Sarah Hatem, Janet C Long, Stephanie Best, Zoe Fehlberg, Bróna Nic Giolla Easpaig, Jeffrey Braithwaite

**Affiliations:** 1 Australian Institute of Health Innovation Macquarie University North Ryde Australia; 2 Australian Genomics Murdoch Children’s Research Institute Melbourne Australia

**Keywords:** mobile apps, self-management, social support, rare disease, mobile phone

## Abstract

**Background:**

Mobile apps are becoming increasingly popular, with 5.70 million apps available in early 2021. Smartphones can provide portable and convenient access to health apps. Here, we consider apps for people with one of the estimated 7000 rare conditions, which are defined as having an incidence of <1 in 2000. The needs of people with rare conditions are known to be different from those of people with more common conditions. The former may be socially isolated (not knowing anyone else who has the condition) and may not be able to find reliable information about the disorder.

**Objective:**

The aim of this review is to search for apps developed specifically for people diagnosed with a rare disease and to assess them for quality using the Mobile App Rating Scale (MARS). We examine features that address 6 identified needs of people with a rare disorder and make recommendations for future developers.

**Methods:**

Google Play Store (Android) and Apple App Store (iOS) were searched for relevant health-related apps specifically for rare diseases. The search included the names of 10 rare disease groups. App quality was determined using MARS, assessing app engagement, functionality, aesthetics, and information.

**Results:**

We found 29 relevant apps (from a total of 2272) addressing 14 rare diseases or disease groups. The most common rare conditions addressed were cystic fibrosis (n=6), hemophilia (n=5), and thalassemia (n=5). The most common app features were web-based information and symptom trackers. The mean MARS score was 3.44 (SD 0.84). Lowest scores were for engagement.

**Conclusions:**

Most apps provided factual and visual information, providing tools for self-monitoring and resources to help improve interactions during health consultations. App origin and quality varied greatly. Developers are recommended to consider ways to make appropriate apps more easily identifiable to consumers, to always include high-quality information, improve engagement, provide qualitative evaluations of the app, and include consumers and clinicians in the design.

## Introduction

### Background

The number of smartphones is growing globally (6.259 million in 2021 and is expected to rise to an estimated 7.690 million by 2027 [1]). Downloadable apps are also growing exponentially, with a total of 5.70 million apps available in early 2021 from the 2 largest app stores, Google Play and the Apple App Store [2]. The ubiquity of mobile phones and their portability mean that apps to support health are convenient and accessible tools for large parts of the population [3]. Health apps can support healthy behaviors (eg, physical exercise [4], mindfulness [5]), support people with a specific condition (eg, diabetes [6], depression and anxiety [7]), or support health needs at certain life stages (eg, prenatal genetic testing [8], healthy aging [9]).

Rare diseases are a heterogenous group of conditions that are defined as having an incidence of <1 in 2000 [10,11]. An estimated 6000 to 8000 rare diseases have been discovered to date, affecting between 3.5% and 8% of the global population [11]. Rare diseases vary in origin and characteristics, including genetic conditions, infectious diseases, autoimmune diseases, and rare cancers [10,11]. Rare diseases are often harder to diagnose than commonly occurring diseases [12]. Although the importance of optimizing care for individuals with rare conditions is widely acknowledged, patients and their families report substantial barriers to accessing high-quality care and support following diagnosis [13]. Individuals living with a rare disease frequently report a lack of access to appropriate health care services, skilled health professionals, and management options [11]. Efforts to provide high-quality care are hampered by scarce research and medical knowledge (with sample sizes too low to run clinical trials for any individual disease), limited treatment options, and a lack of standardized guidelines for clinical management. For many rare diseases, health professionals may only see one case during their entire career [14].

Several studies have reported that people with rare conditions have differing needs from those with high-incidence conditions (eg, [15-17]). Although high-prevalence disorders may be no less distressing or onerous to care for, rare diseases have unique features: the lengthy odyssey to find a diagnosis, then to find appropriate specialists who know about the disorder; the lack of evidence about effective treatments, guidelines, or access to knowledgeable general health service providers; and isolation from peer support. Apps and other eHealth interventions (eg, telehealth, interactive websites, instant messaging, and web-based monitoring) may provide useful tools for health education, disease management, and patient advocacy in this cohort of patients but are likely to be different from apps designed for more common conditions.

This study complements a parallel study by the same authors (unpublished) looking at the needs of people with rare conditions that can be addressed by a range of eHealth tools including apps. That review found there to be 4 domains and 23 subdomains of the needs of people with a rare condition that could be addressed with eHealth interventions. The domains of need were support for self-management, access to high-quality information, access to appropriate specialist services, and social support.

App quality, especially from the viewpoint of consumers choosing an app from a vendor, has been explored and found to be lacking in a number of reviews of apps targeting high-incidence conditions (eg, [8,18,19]) and rare conditions [11]. Frequently mentioned are poor or absent reporting of trialing of the app or the evidence base on which the app is built [8] and difficult to understand information [11,20]. Moreover, the unique needs of people with rare diseases may mean that apps are not being designed to address those needs appropriately and acceptably. Reviews that identify unmet needs and highlight concerns regarding quality are therefore important.

### Objectives

The aim of this review was to scope the nature and range of mobile apps developed specifically for patients diagnosed with a rare disease, or carers or parents of these patients. The Mobile App Rating Scale (MARS) [3] was used to assess the apps. The research questions are (1) What apps are available for people living with a rare disease? and (2) How do apps address reported needs and contribute to appropriate, high-quality care for people living with rare diseases?

To our knowledge, this is the first review of apps for people living with rare diseases. This review will focus on the specific needs of people with rare conditions, identifying the various benefits and shortcomings of existing apps, and will help inform the development of future apps for this often-overlooked group.

## Methods

### Search Strategy

The 2 most popular commercial app stores [2], Google Play Store (Android) and Apple App Store (iOS) were used to search for relevant apps. Searches were run in July 2021 using the Google Chrome feature, Google *incognito* mode, to lessen the influence of searchers’ browser history. The 2 app stores were searched independently by 2 reviewers (SH and BNGE), where BNGE accessed the Google Play Store and SH accessed the Apple App Store using combinations of keywords: “Rare disease” AND “patient education” OR “health resource” OR “delivery of health care” OR “patient advocacy” OR “patient participation” OR “patient resource” OR “rare disease patient.” This approach yielded few apps mostly because of the vagueness of the term “rare disease” (a term that covers 6000-8000 different conditions). The search was then modified to name 10 of the more common rare diseases or disease groups: cystic fibrosis, cystinosis, Fabry disease, hemophilia, hereditary angioedema, mitochondrial disease, narcolepsy, primary biliary cholangitis, spina bifida, and thalassemia (alpha and beta) [21].

### App Selection

The inclusion criteria applied to the apps were (1) focus on a single rare disease or a group of rare diseases, with an incidence of <1 in 2000; (2) targeted at the patient or carer; (3) available in English; and (4) free to download. Apps were excluded if they (1) were directed solely at health professionals, (2) were solely collecting data from patients for research purposes, (3) required a personalized access code or the user had to be in a specific geographic location to create an account, or (4) failed basic functional criteria such as download (after 2 attempts).

First, apps were screened by app name and basic description in the web-based Apple Store and Google Play Store by the authors SH and BNGE (who each performed 20% independently and then compared and discussed). Relevant apps were cataloged in a spreadsheet, and duplicates were removed by the same authors. Subsequently, the apps were formally screened on the web-based store’s site by app name and description against the selection criteria by SH and ZF. Finally, the apps were downloaded on the personal phone devices of SH and ZF, and their features were examined to verify their inclusion. This was validated by the rest of the research team.

### Data Extraction

For the included apps, the following information was extracted (following section 1 of the MARS tool described in the *Quality Approval* section below): app name, name of the rare disease or disease group, platform (iOS or Android), version, year of the latest update, language options, app developer, country of origin, age group of target audience, and purpose or aim. This information was identified from the *General description* features of each app. We further extracted details of app features, whether the app was consumer facing (for the person with the condition or their carer only) or collaborative (to be used with a health care professional) and whether the app required passive or active participant involvement to use (ie, passive requires no interaction or involvement from an individual [eg, general information] and active requires consumer interaction [eg, entering symptoms into a symptom tracker feature]).

Domains of needs of people with a rare disease were defined initially based on a separate review of the peer-reviewed literature that considered the use of all eHealth interventions, not just apps. The four domains of need found and examined were (1) social support, (2) access to high-quality information about their specific rare disease, (3) support for self-management, and (4) access to appropriate specialist services for their rare disease. As the data extraction proceeded for the apps in this review, this list was amended. Domain 4, access to appropriate specialist services for their rare disease, was deleted as not being present in the reviewed apps, and 2 additional domains were added: sharing patient data with the health team and contributing to a global research database or registry. This is shown in Table 1.

Following data extraction, the included apps were examined based on their features and classified according to their domains of need. This classification allowed us to compare the quality of apps with a similar purpose and determine whether apps were addressing the needs of people living with a rare disease.

**Table 1:** Domains of needs of people with a rare disease that may be addressed by apps.

Domain	Examples of app features
Domain 1: social support	Platform where people with the same rare disease can exchange experiences and information (may include input from a health care professional).
Domain 2: tools for improving consults with health professionals	Feature to prerecord questions and record the consultation, advice on setting goals, and so on.
Domain 3: high-quality information on rare disorders	Information on rare disease, negotiating with school or workplaces, up-to-date information on research and new clinical trials, guidelines, and links to appropriate websites.
Domain 4: self-management support	Symptom trackers, journals, medication reminders, appointment reminders, guidance for performing exercise or treatments (may include ability to share inputted data with health care professional).
Domain 5: improve coordination of care	Feature allows sharing of inputted data with multiple health care professionals.
Domain 6: contributing to a global research database or registry	Data can be entered by app user to contribute to global research.

### Quality Appraisal

App quality was assessed using MARS, a tool specifically designed for rating and examining the quality of mobile apps used for health [3]. The tool consists of 6 categories. The first deals with classification (version, developer, targeted age group, etc). The second section rates the objective quality of the apps by assessing 4 attributes: *Engagement* (5 items), *Functionality* (4 items), *Aesthetics* (3 items), and *Information* (7 items). *Engagement* refers to whether the app is fun, interesting, customizable, or interactive (eg, it sends alerts, messages, reminders, enables sharing, and is well targeted to the audience). *Functionality* assesses app functioning, ease of use, navigation, flow logic, and gestural design of the app. *Aesthetics* assesses graphic design, overall visual appeal, color scheme, and stylistic consistency. *Information* assesses whether the apps contain high-quality information and references from a credible source. An example of the scoring criteria is given in Textbox 1 [3].

Scores for each section (3-7 criteria each) are computed as mean scores to allow for criteria that are not applicable (eg, criterion that ask about the quality of the information given in the app, but there is no information).

The last 2 sections of the MARS tool assess the subjective quality of the app and the information on the perceived impact of the app on the user. The authors’ lack of familiarity with the conditions the apps addressed led to the decision to consider only objective criteria.

In total, 19 items were rated on a 5-point scale from 1 *Inadequate* to 5 *Excellent* and combined to create an overall objective quality score. Authors SH and ZF assessed the quality of each app, rated an initial 50% of the apps in parallel, and confirmed acceptable interrater reliability. The 2 authors discussed and resolved any significant conflicts. Descriptive statistical analyses were performed using STATA (version SE 17; StataCorp).

**Scoring criteria for Aesthetics.**

**Scoring criteria**

- Is the arrangement and size of buttons, icons, menus, or content on the screen appropriate or zoomable, if needed?

1. Very bad design, cluttered, some options impossible to select, locate, see, or read device display not optimized.
2. Bad design, random, unclear, some options difficult to select, locate, see, or read.
3. Satisfactory, few problems with selecting, locating, seeing, or reading items or with minor screen size problems.
4. Mostly clear, able to select, locate, see, or read items.
5. Professional, simple, clear, orderly, logically organized, device display optimized. Every design component has a purpose.

## Results

### Principal Results

**Figure 1:**
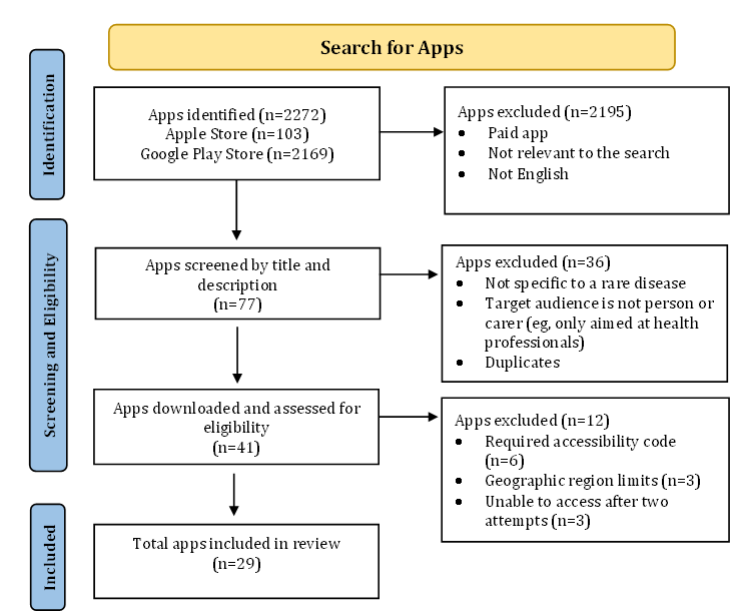
App selection process flowchart.

Our search identified 2272 apps. Following the screening of titles and descriptions in the web-based store, a total of 96.61% (2195/2272) apps were excluded. Most of the excluded apps were not addressing a rare condition. The remaining 77 apps were recorded in a spreadsheet, with duplicates being removed (n=11) and screened again against the inclusion criteria. A total of 41 apps were then downloaded and evaluated against the selection criteria. In all, 29 remained for inclusion. The flow diagram (Figure 1 [22]) provides an overview of the selection process.

### Characteristics of the Apps

Multimedia Appendix 1 summarizes the characteristics of the 29 included apps. Further details are provided in Multimedia Appendices 1 and 2. The apps addressed 14 different rare diseases or rare disease groups. Apps for people with cystic fibrosis were the most commonly identified (6/29, 21%). Our search did not locate any apps for Fabry disease, mitochondrial disease, and hereditary angioedema. Most apps (17/29, 59%) were available on both iOS and Android devices, and most (20/29, 69%) had been updated in the past 2 years. A total of 72% (21/29) of apps were only available in English, and 28% (8/29) were available in multiple languages. Most apps (10/29, 34%) did not specify their country of origin, and the most common developers were not-for-profit organizations or individuals (7/29, 24%). A total of 93% (27/29) of apps targeted consumers aged ≥12 years (2/29, 7% of apps for younger children), 55% (16/29) required active involvement by consumers, and 24% (7/29) were collaborative in nature.

### Features of the Apps

The features of the apps are summarized below. Some apps included more than one feature.

In all, 52% (15/29) of apps included web-based information for patient education, including both factual and visual resources in the form of databases, downloadable information, informative videos, illustrations, and fact sheets. They described general disease information about the rare condition, disease history, signs and symptoms, management, and epidemiology where known. Exemplars are *Recognize Thalassemia Disease* and *Cystic Fibrosis: A Pocket Guide*. The *VASCERN app* contained the latest contact details for each relevant rare disease health care professional and patient organization, including services they provided, and hospital or clinic location information.

A total of 24% (7/29) of apps contained a feature that allowed them to export data trends they had recorded via symptom trackers and digital journals into a file that could then be shared with a health care professional during consultations (eg, *RareGuru* and *Cystic Fibrosis Manager)*. A total of 28% (8/29) of apps included a symptom tracker whereby individuals could record their condition-specific symptoms and side effects (eg, *Narcolepsy Monitor* and *Cystic Fibrosis Manager)*. A total of 24% (7/29) of apps involved the creation of a web-based profile where individuals could connect with others who were diagnosed with the same rare disease. Creating a profile allowed users to share resources, support each other, and stay updated about the latest news and research from different groups and foundations (eg, *RarePulse* and *RareGuru)*. A total of 24% (7/29) of apps included a digital journal, allowing users to record their experiences and feelings as their condition progressed, likely to be useful in helping individuals during consultations with health care professionals (eg, *PBC Health Storylines* and *Cystic Fibrosis Manager)*. Other features included medication reminders (5/29, 17%), medical appointment reminders (3/29, 10%; eg, *ThalTracker*), and the option to record medical tests (3/29, 10%). This last feature could be accessed by both health care professionals and individuals with the option to store the results in the patient profile (*PBC Health Storylines* and *Cystic Fibrosis Manager)*.

A total of 7% (2/29) of apps, *Breathe RM* and *PBC Health Storylines*, included the option to sync with a wearable device such as Fitbit or Apple Watch and allowed the app to access further information about vital signs and heart rate. The *Autogenic Drainage* app featured treatment support and *Cystic Fibrosis Downhill* featured an interactive game for children and adults as a form of patient education regarding their condition.

### Quality Appraisal of the Apps

The total mean score of the 29 apps across the 4 MARS quality domains was, on a 5-point scale, 3.44 (SD 0.84). *Narcolepsy Monitor* had the maximum score at 4.69 and *Thalassemia Disease* had the minimum score at 1.95. The *Functionality* domain had the highest mean 4.23 (SD 0.62) across all apps and had the smallest variation in minimum and maximum scores (3.25 to 5). This means that navigation, ease of use, performance, and gestural design (eg, swipes and taps) across the apps were mostly intuitive and well designed. The lowest mean score was for the *Engagement* domain 2.94 (SD 1.08), which had the highest variation between minimum and maximum (1.2 to 4.8). For example, one of the lowest scoring apps for *Engagement* was *Autogenic Drainage*, which leads the user through a deep breathing exercise. Although it has some customizable features (duration of the session) and some encouraging messages (“Try not to cough!”), there are no graphics—only text and a basic timer—limiting its ability to engage.

The *Information* section of the MARS tool had the second lowest mean scores (second to *Engagement)*, reflecting deficits in quality, conciseness, ease of understanding, and use of the evidence base. One of the items in the information section of the MARS tool asks whether the “App has been trialed or tested and must be verified by evidence (in published scientific literature).” All the apps scored 0 for this item. Table 2 provides the mean scores across the 4 MARS quality domains and the total mean score, ranking from highest to lowest.

**Table 2:** App quality mean scores across the 4 Mobile App Rating Scale sections and total (out of 5).

		Engagement	Functionality	Aesthetics	Information	Total
Overall mean (SD; range)		2.94 (1.08; 1.2-4.8)	4.23 (0.62; 3.25-5)	3.40 (1.25; 1.33-5)	3.23 (0.98; 1.5-4.6)	3.45 (0.84; 1.95-4.69)
**App name**						
	Narcolepsy Monitor	4.80	4.75	4.70	4.50	4.69
	Cure SMA guide	4.60	5.00	4.70	4.40	4.68
	Cystic Fibrosis Manager	4.60	4.75	4.70	4.00	4.51
	Project Breathe or Breathe RM	3.80	4.75	5.00	4.00	4.39
	RarePulse	4.40	4.75	3.67	4.60	4.36
	PatientMpowerment for CF	3.80	5.00	4.67	3.75	4.30
	RareGuru	3.8	4.50	4.33	4.00	4.16
	MicroHealth Hemophilia	3.60	4.25	5.00	3.50	4.09
	Cystinosis & me	4.00	4.50	3.67	4.00	4.04
	Spina Bifida Association	2.40	5.00	4.67	3.83	3.98
	HaemActive	2.60	4.75	5.00	3.50	3.96
	PBC Health Storylines	3.80	4.00	4.00	3.33	3.78
	ThalTracker	2.60	4.25	4.33	3.86	3.76
	CANrecall	2.60	4.25	3.67	4.00	3.63
	THALIA app	3.20	3.75	3.00	4.00	3.49
	VASCERN app	2.20	4.00	3.67	4.00	3.47
	ThaliMe	3.40	3.50	3.67	3.00	3.39
	Cystic Fibrosis Downhill	3.40	4.00	3.67	2.00	3.27
	Cystic Fibrosis: A Pocket Guide	2.40	3.75	3.33	3.17	3.16
	PH Aware	2.80	3.33	2.33	4.00	3.12
	Haemophilia Pal	2.60	4.00	3.00	2.50	3.03
	MyHemophiliaTeam	3.20	3.25	2.67	2.75	2.97
	Autogenic Drainage	2.60	4.00	2.00	2.50	2.78
	Recognize Amyloidosis Disease	1.40	5.00	1.33	2.00	2.43
	Recognize Thalassemia Disease	1.40	5.00	1.33	2.00	2.43
	Easy Diagnosis- Thalassemia	1.20	5.00	1.33	1.60	2.28
	Narcolepsy Disorder	1.40	3.25	1.67	1.67	2.00
	Hemophilia Disease	1.40	3.25	1.67	1.67	2.00
	Thalassemia Disease	1.40	3.25	1.67	1.50	1.95

### Needs That the Apps Address

#### Overview

All 6 domains of need (defined in Table 1) were addressed by at least one of the 29 apps. More than half the apps (17/29, 59%) aimed to address domains 3 (access to high-quality information for their rare disease) and 4 (support for self-management). Less common (3/29, 10%) were apps that aimed to address domain 6 (contributing to a global research database or registry). This may have been owing to a majority of these apps being filtered out in the screening phase, as they were generally targeted at health care professionals and researchers instead of diagnosed individuals. Table 3 provides a summary of the apps and the needs they address. The number of needs each app aimed to address varied. Most apps were identified as addressing 1 or 2 needs (23/29, 79%). A total of 17% (5/29) of apps addressed 3 or 4 domains, and 3% (1/29) of app (*Cure SMA Guide*) aimed to address all 6 needs. In addition, 3% (1/29) of apps provided users with knowledge of how to negotiate or advocate for their needs in non–health care settings (schools, workplaces, or insurance).

**Table 3:** Domains of needs of people with a rare disease addressed by each app.

			Total domains, N=6		Need domains										
					Social support		Tools for improving consults with health professionals		High-quality information on rare disorders		Tools to support self-management		Sharing patient data with the health team or improving coordination of care or both		Facilitate contribution to a global research database or registry
Total			54		4		7		17		17		7		3
**App name**															
	Narcolepsy Monitor	2				✓				✓		✓			
	Cure SMA guide	6		✓		✓		✓		✓		✓		✓	
	Cystic Fibrosis Manager	4				✓		✓		✓		✓			
	Project Breathe or Breathe RM	1								✓					
	RarePulse	2						✓						✓	
	PatientMpowerment for CF	3								✓		✓		✓	
	RareGuru	2				✓				✓					
	MicroHealth Hemophilia	2								✓		✓			
	Cystinosis & me	2				✓				✓					
	Spina Bifida Association	2						✓		✓					
	HaemActive	2								✓					
	PBC Health Storylines	2								✓		✓			
	ThalTracker	2								✓		✓			
	CANrecall	2				✓				✓					
	THALIA app	3				✓		✓		✓					
	VASCERN app	2		✓				✓							
	ThaliMe	2		✓						✓					
	Cystic Fibrosis Downhill	1						✓							
	Cystic Fibrosis: A Pocket Guide	1						✓							
	PH Aware	1						✓							
	Haemophilia Pal	1								✓					
	MyHemophiliaTeam	2		✓				✓							
	Autogenic Drainage	1								✓					
	Recognize Amyloidosis Disease	1						✓							
	Recognize Thalassemia Disease	1						✓							
	Easy Diagnosis- Thalassemia	1						✓							
	Narcolepsy Disorder	1						✓							
	Hemophilia Disease	1						✓							
	Thalassemia Disease	1						✓							

#### Social Support

A total of 14% (4/29) of apps included characteristics that addressed social isolation. These apps allowed consumers to actively connect and liaise with others with the same condition, as well as others who were part of the general rare disease community. Features of these apps included the ability to create a web-based profile, connect on social media platforms, join community support groups, and take part in web-based forums and social events. These features were evident in *Cure SMA Guide*, the *VASCERN app*, *ThaliMe*, and *MyHemophiliaTeam*.

#### Improving Consults With Health Care Professionals

A total of 31% (9/29) of apps included tools that were targeted at improving communication between a patient and their health care professional or care team. These apps were often collaborative in nature, whereby patients could export their collected data to share with their care teams and health care professionals could connect with their patients via telehealth or direct messaging. Consumers had the opportunity to share real-time condition tracking with their health care professionals on some apps. Health care teams could digitally send and store laboratory results on the app and schedule appointments. These features were seen in *Narcolepsy Monitor, Cure SMA Guide, Cystic Fibrosis Manager, RareGuru, Cystinosis &me, CANrecall*, and *THALIA app*. An app, *CANrecall*, had a list of question prompt lists clinically designed to help individuals ask meaningful questions during a specialist appointment. This app also allowed patients to record their session and listen to their consultation later.

#### Access to High-Quality Information on Rare Disorders

A total of 59% (17/29) of apps included features that aimed to educate the user about a specified rare disease and provided access to a range of detailed information. This allowed the diagnosed individuals or their carers to expand their knowledge by reading reliable sources such as diagnosis, treatment, and management of the condition. Some apps offer these in an easy-to-read PDF format, video, illustrations, through external links, or questionnaires. This was displayed in *Cure SMA Guide, RarePulse, Narcolepsy Disorder, Cystic Fibrosis: A Pocket Guide, Spina Bifida Association*, and others (Table 3). An app, *Cystic Fibrosis Downhill*, creatively provided this using an interactive educational game. Others included the latest contact and location details of health care providers and patient organizations as well as the services they provided. The *Cure SMA Guide* included care guidelines, treatments, patient and hospitalization guidelines, and sample letters for emergency resources. These sample letters helped the individual liaise with hospitals, physicians, insurers, electricity billing companies, telephone providers, schools, and emergency departments.

#### Self-management Support

A total of 59% (17/29) of apps incorporated characteristics that enabled better self-management of the condition. Apps that met these characteristics commonly included symptom trackers, options for medication reminders and appointments, and export data trends for sharing with physicians and specialists. These apps ranged from patient facing only to collaborative, allowing access from both the health care team and the patient. Examples of these features were in *Narcolepsy Monitor*, *Cure SMA Guide*, and *Breathe RM*. Remote monitoring was also available in the *HaemActive* app, where patients could participate in exercises on their own or in consultation with their physiotherapist via a video function. Remote monitoring was also available in the *PatientMpowerment for CF* app.

#### Improve Coordination of Care

A total of 24% (7/29) of apps included features for improving the coordination of care between health care physicians and teams, allowing for collaborative participation. Members of the multidisciplinary team could log on to the app and update any important information such as previous medicine treatments and test results; for example, *Cystic Fibrosis Manager*.

#### Contributing to a Global Research Database or Registry

A total of 7% (2/29) of apps gave the consumer the opportunity to share their deidentified data to help contribute to global research for their condition, supporting the development of a new understanding of treatments for the disease. These were *Cure SMA Guide* and *PatientMpowerment for CF*. An app, *Rare Pulse*, provided features that helped patients and caregivers stay in the loop about the latest news and updates from different research groups and consumer advocacy agencies, while furnishing information on upcoming forums and events regarding the rare disease condition.

## Discussion

### Principal Findings

Apps have the potential to enhance the quality of care, management of care, and self-management for individuals diagnosed with a rare disease [23] and can contribute to patient empowerment in an area of health care that can be confusing to navigate. People with a rare disorder face unique barriers to accessing appropriate care, including social isolation, difficulty in comprehending health care practitioner communications, lack of information, complicated self-management, poor coordination of care between health teams, and obstacles in accessing relevant research. High-quality apps have the potential to provide cohesive and trustworthy information, tools to collect symptom and treatment data, and options to assist in liaising with health professionals during consultations.

This review found only a small number of health apps targeting rare conditions. There is the obvious problem that a low number of potential users is a poor incentive for developing an app, especially a commercial app (eg, in 2019, 28.7 million people in the United States had diabetes [24] compared with 35,000 people who had cystic fibrosis [25]). Apps for these rarer conditions, therefore, tend to be developed and funded by not-for-profit organizations—patient advocacy agencies or clinician-research collaborations. We note that 55% (16/29) of the apps found in this review were developed by not-for-profit patient organizations or research groups.

Searching for apps for people with a rare disorder highlighted 2 sets of issues. First, for us as researchers, using the term “rare disease” yielded few results. Adding selected named rare disease groups increased this yield; however, an exhaustive search was not feasible. Rare disease group names are an inexact way to search. For example, there were no apps found for “mitochondrial disease.” This rare disease group name covers over 350 different disorders, with names that use medical language or refer to the gene that is faulty (eg, DNM1L-related encephalopathy and Leber hereditary optic neuropathy). Moreover, many of these disorders go by several different names (eg, “MELAS” for ORPHA-550, OMIM 540000, mitochondrial encephalomyopathy lactic acidosis and stroke-like episodes or mitochondrial myopathy encephalopathy lactic acidosis and stroke-like episodes [26]). It was not feasible to consider a search for all these alternatives across all rare disorders.

Second, people with a rare disease may have difficulty finding relevant apps by searching by diagnosis. Precisely naming an app (eg, “My DNM1L-related encephalopathy”) is an unlikely choice for developers, who are more likely to use a more appealing, colloquial name. Therefore, consumers may need to search for the disease group (eg, genetic disorders, neurological disorders, or brain disorders). This suggests that more thought should be given by developers to tag or label their apps so they can be easily found in consultation with clinical experts.

Many of the apps found in our review (16/29, 21%) provided some sort of web-based information about the disease, but as noted earlier, the *Information* items of the MARS received the second lowest mean scores of the 4 sections. Access to information from a reliable source is a requirement for people with rare conditions. Apps targeting people with rare diseases that do not contain information should seriously consider adding this function. The inclusion of reputable, high-quality, and easy-to-understand information and guidelines (where available) is recommended for all health apps [27]. For rare disease apps particularly, including links to internationally recognized rare disease information sites (eg, Orphanet) would be a useful addition.

We found no evidence in peer-reviewed literature that any app was formally tested for usability. Other reviews of apps for higher-prevalence conditions also found deficits in the information section of the MARS scale. For example, a review of apps for women undergoing prenatal testing found an absence of developmental testing with end users. In other items in the same review, in the information section, they found missing, incorrect, or difficult to understand information on the tests and a lack of visual information. We note that large-scale quantitative testing of the apps in our review may not be feasible because of the low number of users, but we suggest that qualitative evaluation is an acceptable alternative.

The impact of health apps may be constrained by their engagement with users. Loss of interest in using health apps over time is well documented (eg, [28]). The MARS tool scores for this review appeared similar in means and ranges across the respective sections of other reviews of health apps (eg, [3,29]). The lowest scoring section of the 4 MARS domains was for *Engagement*. This is a clear area in need of improvement. High-quality apps with simple functionality can encourage people with no prior experience using technology to embrace their use [29]. Interesting visuals and interactive functions are useful components for increasing users’ desire to engage with an app [27,29].

Some apps incorporated active participation by users in the form of digital journals, medication reminders, appointment reminders, and capacity for recording test results. Symptom trackers (8/29, 28%) were the second most common feature of the apps and were noted to be more common than treatment facilitators (1/29, 3%). This likely reflects the lack of available treatment for many rare diseases. Self-assessments via smartphones can save time during a consultation and allow the patient to provide health care professionals with a more accurate update on their condition and improve their approach and confidence to engage in self-management practices [3].

Formal evaluation of clinical outcomes supported by the apps is desirable, but we found little evidence to support this. Only one app alluded to an evaluation in a clinical study, but the details could not be identified in PubMed or Google Scholar. Lack of clinical testing similarly reflects the lack of agreed clinical indicators, treatments, or sufficient numbers of patients to participate in trials in the field of rare diseases.

### Strengths and Limitations

A strength of this paper includes the use of the high-quality appraisal tool, MARS [3]. The method used here to scope and assess apps is generalizable to other health conditions. Limitations were around the problems of searching for >7000 types of rare diseases. Results were limited by access to some apps; for example, ones that required an access code from an external source or required payment. The data security check could not be performed comprehensively with the available data.

### Recommendations

This review suggests a number of recommendations for developers of apps for people with a rare disease: (1) appropriate apps that address rare conditions can be hard to find, so developers should carefully consider how to make their app easily identifiable in the app stores; (2) developers should consider the needs of people with rare conditions when developing apps and not just follow designs used by high-incidence conditions. In particular, the need for high-quality information and social support (ie, consumers may not know anyone else who has the condition) should be considered. Information should be sourced from high-quality sources and checked with clinicians who specialize in the disorder; (3) low scores found in this review for the MARS *Engagement* criteria argue for more thought being directed to designing interesting and engaging features; (4) the subjective star rating system is not always helpful for apps for people with a rare disease. Although the star system may be helpful for consumers trying to choose between 50 different apps for diabetes, most of the apps for rare diseases in this review had no rating or had only a handful of users each. Usability testing and other quality ratings of apps should be considered, as well as formal qualitative evaluations; and (5) following the expression “Nothing about us, without us” [30], the input of consumers via advocacy agencies (or as individuals) is important to develop a feasible, credible, and useful app.

### Conclusions

This review aimed to identify and evaluate mobile apps in the Apple App Store and Google Play Store that addressed the needs of people diagnosed with a rare disease. Most apps focused on providing factual and visual information, tools for monitoring symptoms and resources to help improve interactions during health consultations. App quality and origin varied significantly. Developers are encouraged to consider the unique needs of people with a rare condition to make appropriate, engaging, easy-to-find, and useful apps for this often-neglected cohort.

## Appendix

Multimedia Appendix 1 General characteristics of the included apps. Level of participant interaction involved in the app, for example, information only (passive) or more of a hands-on resource (active), for example, symptom trackers.

Multimedia Appendix 2 Details of the included Apps.

## Abbreviations

MARS: Mobile App Rating Scale
