# Supplementary material for: Mobile Apps for People With Rare Diseases: Review and Quality Assessment Using Mobile App Rating Scale
Source: J Med Internet Res. 2022 Jul 26;24(7):e36691. doi: 10.2196/36691 (PMC9364167; doi:10.2196/36691)
Supplement: Multimedia Appendix 1 [file jmir_v24i7e36691_app1.pdf]

Multimedia Appendix 1: General information about apps.

[a]Level of participant interaction involved in the app, for example Information only (passive) or more of a hands-on resource (active) for example symptom trackers.

App characteristics (N=29)	
	**Frequency, n (%)**
**Rare disease**	
Amyloidosis Disease	1 (4)
Cystic fibrosis	6 (21)
Cystinosis	1 (4)
Hemophilia	5 (17)
Multiple rare diseases	2 (7)
Narcolepsy	2 (7)
Primary Biliary Cholangitis	1 (4)
Pulmonary Hypertension	1 (4)
Rare cancers	1 (4)
Rare vascular diseases	1 (4)
Sickle cell disease	1 (4)
Spina Bifida	1 (4)
Spinal muscular atrophy	1 (4)
Thalassemia (alpha and beta)	5 (17)
**Platform**	
iOS only	2 (7)
Android only	10 (35)
iOS and Android	17 (59)
**Year of latest update**	
2013	1 (4)
2014	1 (4)
2015	1 (4)
2016	1 (4)

2017	5 (17)
2018	0 (0)
2019	4 (14)
2020	5 (17)
2021	11 (38)
**Languages**	
English only	21 (72)
English and other	8 (28)
**Country**	
Australia	1 (4)
Canada	3 (10)
Denmark	1 (4)
International	2 (7)
Netherlands	1 (4)
UK	2 (7)
USA	8 (28)
USA and Europe	1 (4)
Not recorded	10 (35)
**Developer**	
App developer	7 (24)
Clinician	3 (10)
Consumer	3 (10)
For-profit-organization	6 (21)
Not-for-profit organization	7 (24)
Not-for-profit organization and consumers	1 (4)
Researchers	1 (4)
Researchers and universities	1 (4)
Universities and consumers	1 (4)
**Target age group, years**	
<4	1 (4)

<9	1 (4)
<12	6 (21)
<17	8 (28)
All ages	13 (45)
**Target audience**	
Person with condition only	14 (48)
Person with condition and carer	15 (52)
**Participant involvement[a],**	
Passive	9 (31)
Active	16 (55)
Passive and Active	4 (4)
**Collaboration, n (%)**	
Consumer facing only	12 (41)
Collaborative with health professional	7 (24)
Consumers and health care professionals can use it independently	10 (35)
