# Supplementary material for: Mobile Apps for People With Rare Diseases: Review and Quality Assessment Using Mobile App Rating Scale
Source: J Med Internet Res. 2022 Jul 26;24(7):e36691. doi: 10.2196/36691 (PMC9364167; doi:10.2196/36691)
Supplement: Multimedia Appendix 2 [file jmir_v24i7e36691_app2.pdf]

Multimedia Appendix 2: Details of included Apps

Note: [1]Whether or not an app allows for patient and health care practitioner collaboration; [2] Other languages (Bulgarian, Czech, Finnish, French, German, Greek, Hungarian, Indonesian, Italian, Japanese, Korean, Malay, Polish, Portuguese, Romanian, Russian, Chinese, Spanish, Swedish, Thai, Turkish, Ukrainian, Vietnamese, Welsh); [3] Not recorded

| **Name of the app** | **Rare disease (age group)** | **Platform** | **Country** | **Affiliations** | **Participant involvement (Passive or Active)** | **Target audience (person/carer or both)** | **Collaboration Consumer facing only or collaborative[1]** | **Rating** | **Version** | **Last update** | **Cost** |
|---|---|---|---|---|---|---|---|---|---|---|---|
| Narcolepsy Monitor | Narcolepsy (17+) | iOS Android | Netherlands | NGO University | Active | Person | Consumer-facing only | Hasn't received enough ratings or reviews to receive a summary | 1.0.2 | 2020 | Free |
| Cure SMA guide | Spinal muscular atrophy (12+) | iOS Android | USA | NGO | Both | Both | Consumers & HCPs can use independently | Hasn't received enough ratings or reviews to display a summary | 2021.1 | 2021 | Free |
| Cystic Fibrosis Manager | Cystic fibrosis (17+) | iOS | USA | Commercial | Both | Person | Collaborative | Hasn't received enough ratings or reviews to display a summary | 10.8.0 | 2022 | Free |
| Project Breathe/Breathe RM | Cystic fibrosis (12+) | iOS Android | UK | Commercial | Active | Person | Consumer-facing only | Hasn't received enough ratings or reviews to | 3.8.0 | 2022 | Free |

| | | | | | | | | | | | |
|---|---|---|---|---|---|---|---|---|---|---|---|
| | | | | | | | | display a summary | | | |
| RarePulse | Multiple rare diseases (all ages) | iOS Android | NR[3] | NGO | Passive | Both | Consumers & HCPs can use independently | Hasn't received enough ratings or reviews to display a summary | 1.1 | 2020 | Free |
| PatientMpowerment for CF | Cystic fibrosis (17+) | iOS Android | USA Europe | NGO | Active | Person | Collaborative | Hasn't received enough ratings or reviews to display a summary | 1.7.0 | 2022 | Free |
| RareGuru | Multiple rare diseases (12+) | iOS Android | USA | NGO | Active | Both | Collaborative | Hasn't received enough ratings or reviews to display a summary | 1.2.6 | 2021 | Free |
| MicroHealth Hemophilia | Haemophilia (17+) | iOS Android | NR | Commercial | Active | Both | Consumers & HCPs can use independently | Hasn't received enough ratings or reviews to display a summary | 3.8.8 | 2022 | Free |
| Cystinosis & me | Cystinosis (12+) | iOS Android | USA | Commercial | Active | Person | Collaborative | Hasn't received enough ratings or reviews to display a summary | 0.15.1 | 2022 | Free |
| Spina Bifida Association | Spina bifida (17+) | iOS Android | USA | NGO | Both | Person | Consumers & HCPs can use independently | Hasn't received enough ratings or reviews to | 1.0.47 | 2020 | Free |

| | | | | | | | | | | | |
|---|---|---|---|---|---|---|---|---|---|---|---|
| | | | | | | | | display a summary | | | |
| HaemActive ™ | Haemophilia (12+) | iOSAndroid | Denmark | Commercial | Active | Person | Collaborative | Hasn't received enough ratings or reviews to display a summary | 1.3.3 | 2022 | Free |
| PBC Health Storylines | Primary biliary cholangitis (all ages) | Android | Canada | NGO | Active | Person | Consumer-facing only | Hasn't received enough ratings or reviews to display a summary | 5.4.4 | 2017 | Free |
| ThalTracker | Thalassaemia (12+) | iOS | Canada | NGO University | Active | Person | Consumer-facing only | Hasn't received enough ratings or reviews to display a summary | Unknown | Unknown | Free |
| CANrecall | Rare cancers (17+) | iOSAndroid | Australia | Commercial | Active | Person | Collaborative | Hasn't received enough ratings or reviews to display a summary | 2.2 | 2022 | Free |
| THALIA app | Thalassaemia & sickle cell disease (17+) | iOSAndroid | International | Commercial | Active | Person | Consumer-facing only | Hasn't received enough ratings or reviews to display a summary | 1.1 | 2021 | Free |
| VASCERN app | Rare vascular diseases (all ages) | iOSAndroid | International | NGO | Passive | Both | Consumers & HCPs can use independently | Hasn't received enough ratings or | 1.0.9 | 2022 | Free |

| | | | | | | | | | | | |
|---|---|---|---|---|---|---|---|---|---|---|---|
| | | | | | | | | reviews to display a summary | | | |
| ThlaliMe | Thalassaemia (all ages) | iOS Android | Canada | NGO | Active | Both | Consumer-facing only | Not stated | 3.8.0 | 2019 | Free |
| Cystic Fibrosis Downhill | Cystic fibrosis (all ages) | Android | USA | Commercial | Active | Both | Consumer-facing only | Not stated | 1.0 | 2016 | Free |
| Cystic Fibrosis: A Pocket Guide | Cystic fibrosis (all ages) | Android | UK | NGO | Passive | Both | Consumers & HCPs can use independently | 3.8 | 1.0.3 | 2013 | Free |
| PH Aware | Pulmonary hypertension (4+) | iOSAndroid | NR | NGO | Passive | Both | Consumers & HCPs can use independently | Hasn't received enough ratings or reviews to display a summary | 1.278.54 1.1101 | 2020 | Free |
| Haemophilia Pal | Haemophilia (all ages) | Android | NR | NGO | Active | Person | Collaborative | Not stated | 1.8.3 | 2019 | Free |
| MyHemophiliaTeam | Haemophilia (17+) | iOS Android | USA | Commercial | Active | Both | Consumer-facing only | Hasn't received enough ratings or reviews to display a summary | 12.0.8 | 2020 | Free |
| Autogenic Drainage | Cystic fibrosis (9+) | iOS Android | USA | Commercial | Active | Person | Consumer-facing only | Hasn't received enough ratings or reviews to display a summary | 1.2 | 2015 | Free |
| Recognize Amyloidosis Disease | Amyloidosis disease (all ages) | Android | NR | Commercial | Passive | Both | Consumers & HCPs can use independently | Hasn't received enough ratings or reviews to display a summary | 3.0.1 | 2019 | Free |

| | | | | | | | | | | | |
|---|---|---|---|---|---|---|---|---|---|---|---|
| Recognize Thalassemia Disease | Thalassaemia (all ages) | Android | NR | Commercial | Passive | Both | Consumers & HCPs can use independently | Hasn't received enough ratings or reviews to display a summary | 3.0.1 | 2019 | Free |
| Easy Diagnosis-Thalassemia | Thalassaemia (all ages) | Android | NR | Commercial | Both | Person | Consumer-facing only | Hasn't received enough ratings or reviews to display a summary | 1.0 | 2021 | Free |
| Narcolepsy Disorder | Narcolepsy (all ages) | Android | NR | NGO | Passive | Both | Consumers & HCPs can use independently | Hasn't received enough ratings or reviews to display a summary | Not stated | 2017 | Free |
| Hemophilia Disease | Haemophilia (all ages) | Android | NR | Commercial | Passive | Both | Consumer-facing only | Hasn't received enough ratings or reviews to display a summary | Not stated | 2017 | Free |
| Thalassemia Disease | Thalassaemia (all ages) | Android | NR | Commercial | Passive | Both | Consumer-facing only | Hasn't received enough ratings or reviews to display a summary | Not stated | 2017 | Free |
